# GAA-*FGF14* disease: defining its frequency, molecular basis, and 4-aminopyridine response in a large downbeat nystagmus cohort

**DOI:** 10.1016/j.ebiom.2024.105076

**Published:** 2024-03-19

**Authors:** David Pellerin, Felix Heindl, Carlo Wilke, Matt C. Danzi, Andreas Traschütz, Catherine Ashton, Marie-Josée Dicaire, Alexanne Cuillerier, Giulia Del Gobbo, Kym M. Boycott, Jens Claassen, Dan Rujescu, Annette M. Hartmann, Stephan Zuchner, Bernard Brais, Michael Strupp, Matthis Synofzik

**Affiliations:** a Department of Neurology and Neurosurgery, Montreal Neurological Hospital and Institute, McGill University, Montreal, QC, Canada; b Department of Neuromuscular Diseases, UCL Queen Square Institute of Neurology and the National Hospital for Neurology and Neurosurgery, University College London, London, United Kingdom; c Department of Neurology and German Center for Vertigo and Balance Disorders, University Hospital, Ludwig-Maximilians University, Munich, Germany; d Division Translational Genomics of Neurodegenerative Diseases, Hertie-Institute for Clinical Brain Research and Center of Neurology, University of Tübingen, Tübingen, Germany; e German Center for Neurodegenerative Diseases (DZNE), Tübingen, Germany; f Dr. John T. Macdonald Foundation Department of Human Genetics and John P. Hussman Institute for Human Genomics, University of Miami Miller School of Medicine, Miami, FL, USA; g Department of Neurology, Royal Perth Hospital, Perth, WA, Australia; h Children's Hospital of Eastern Ontario Research Institute, University of Ottawa, Ottawa, ON, Canada; i Department of Neurology, University Hospital Essen, University Duisburg-Essen, Essen, Germany; j MediClin Klinik Reichshof, Reichshof-Eckenhagen, Germany; k Department of Psychiatry and Psychotherapy, Comprehensive Center for Clinical Neurosciences and Mental Health (C3NMH), Medical University of Vienna, Vienna, Austria; l Department of Human Genetics, McGill University, Montreal, QC, Canada; m Centre de Réadaptation Lucie-Bruneau, Montreal, QC, Canada

**Keywords:** SCA27B, GAA-FGF14 ataxia, Downbeat nystagmus, 4-Aminopyridine, Treatment, Trial

## Abstract

**Background:**

GAA-*FGF14* disease/spinocerebellar ataxia 27B is a recently described neurodegenerative disease caused by (GAA)_≥250_ expansions in the fibroblast growth factor 14 (*FGF14*) gene, but its phenotypic spectrum, pathogenic threshold, and evidence-based treatability remain to be established. We report on the frequency of *FGF14* (GAA)_≥250_ and (GAA)_200-249_ expansions in a large cohort of patients with idiopathic downbeat nystagmus (DBN) and their response to 4-aminopyridine.

**Methods:**

Retrospective cohort study of 170 patients with idiopathic DBN, comprising in-depth phenotyping and assessment of 4-aminopyridine treatment response, including re-analysis of placebo-controlled video-oculography treatment response data from a previous randomised double-blind 4-aminopyridine trial.

**Findings:**

Frequency of *FGF14* (GAA)_≥250_ expansions was 48% (82/170) in patients with idiopathic DBN. Additional cerebellar ocular motor signs were observed in 100% (82/82) and cerebellar ataxia in 43% (35/82) of patients carrying an *FGF14* (GAA)_≥250_ expansion. *FGF14* (GAA)_200-249_ alleles were enriched in patients with DBN (12%; 20/170) compared to controls (0.87%; 19/2191; OR, 15.20; 95% CI, 7.52–30.80; *p* < 0.0001). The phenotype of patients carrying a (GAA)_200-249_ allele closely mirrored that of patients carrying a (GAA)_≥250_ allele. Patients carrying a (GAA)_≥250_ or a (GAA)_200-249_ allele had a significantly greater clinician-reported (80%, 33/41 vs 31%, 5/16; RR, 2.58; 95% CI, 1.23–5.41; Fisher's exact test*, p* = 0.0011) and self-reported (59%, 32/54 vs 11%, 2/19; RR, 5.63; 95% CI, 1.49–21.27; Fisher's exact test*, p* = 0.00033) response to 4-aminopyridine treatment compared to patients carrying a (GAA)_<200_ allele. Placebo-controlled video-oculography data, available for four patients carrying an *FGF14* (GAA)_≥250_ expansion, showed a significant decrease in slow phase velocity of DBN with 4-aminopyridine, but not placebo.

**Interpretation:**

This study confirms that *FGF14* GAA expansions are a frequent cause of DBN syndromes. It provides preliminary evidence that (GAA)_200-249_ alleles might be pathogenic. Finally, it provides large real-world and preliminary piloting placebo-controlled evidence for the efficacy of 4-aminopyridine in GAA-*FGF14* disease.

**Funding:**

This work was supported by the Clinician Scientist program “PRECISE.net” funded by the 10.13039/501100003042Else Kröner-Fresenius-Stiftung (to CW, AT, and MSy), the grant 779257 “Solve-RD” from the European’s Union Horizon 2020 research and innovation program (to MSy), and the grant 01EO 1401 by the German 10.13039/501100002347Federal Ministry of Education and Research (BMBF) (to MSt). This work was also supported by the 10.13039/501100001659Deutsche Forschungsgemeinschaft (DFG, German Research Foundation) N° 441409627, as part of the PROSPAX consortium under the frame of EJP RD, the European Joint Programme on Rare Diseases, under the EJP RD COFUND-EJP N° 825575 (to MSy, BB and—as associated partner—SZ), the NIH 10.13039/100000065National Institute of Neurological Disorders and Stroke (grant 2R01NS072248-11A1 to SZ), the Fondation Groupe Monaco (to BB), and the 10.13039/501100017657Montreal General Hospital Foundation (grant PT79418 to BB). The Care4Rare Canada Consortium is funded in part by 10.13039/100008762Genome Canada and the 10.13039/501100000092Ontario Genomics Institute (OGI-147 to KMB), the 10.13039/501100000024Canadian Institutes of Health Research (CIHR GP1-155867 to KMB), 10.13039/100012171Ontario Research Foundation, Genome Quebec, and the Children's Hospital of Eastern Ontario Foundation. The funders had no role in the conduct of this study.

**Research in context**

**Evidence before this study**

Downbeat nystagmus is the most common form of acquired persisting nystagmus, but it remains without aetiological diagnosis (idiopathic) in a substantial number of patients. *FGF14* (GAA)_≥250_ repeat expansions have recently been identified as the cause of GAA-*FGF14* disease/spinocerebellar ataxia 27B, which is a late-onset slowly progressive cerebellar ataxia that is frequently associated with downbeat nystagmus.

**Added value of this study**

This study confirms that *FGF14* (GAA)_≥250_ expansions are a highly frequent cause of downbeat nystagmus syndromes, accounting for 48% of hitherto idiopathic cases, thus establishing downbeat nystagmus syndromes as a common endophenotype of GAA-*FGF14* disease. *FGF14* (GAA)_200-249_ alleles were significantly enriched in patients with downbeat nystagmus compared to controls; furthermore, the phenotype of patients carrying a (GAA)_200-249_ allele was similar to that of patients carrying a (GAA)_≥250_ expansion. 4-aminopyridine improved downbeat nystagmus, gait, and disability in a substantial proportion of patients with GAA-*FGF14* disease.

**Implications of all the available evidence**

Genetic testing for *FGF14* GAA repeat expansions should now become part of the routine diagnostic work-up of patients with idiopathic downbeat nystagmus, especially in the presence of additional cerebellar signs, as they account for a significant proportion of cases. This study provides evidence for the potential pathogenicity of alleles of 200–249 repeats and adds several lines of evidence for the treatment efficacy of 4-aminopyridine in GAA-*FGF14* disease. It further paves the way toward clinical trials of 4-aminopyridine in GAA-*FGF14* disease.

## Introduction

A dominantly inherited GAA repeat expansion in intron 1 of the fibroblast growth factor 14 (*FGF14*) gene has recently been identified as the cause of GAA-*FGF14* disease/spinocerebellar ataxia 27B (SCA27B),^1^^,^^2^ a late-onset slowly progressive cerebellar syndrome that is frequently associated with episodic symptoms and downbeat nystagmus (DBN).^1^^,^^3^, ^4^, ^5^ While this core phenotype has been well delineated,^1^, ^2^, ^3^, ^4^, ^5^, ^6^, ^7^, ^8^ the full clinical spectrum of this disease remains to be determined. Moreover, the pathogenic threshold of *FGF14* GAA expansions, previously proposed to be at least 250 repeat units,^1^^,^^2^ and evidence-based treatability of GAA-*FGF14* disease with 4-aminopyridine (4-AP), which has shown promising benefits to reduce the frequency and severity of ataxic symptoms in small open-label case series,^1^^,^^3^^,^^9^ are yet to be further established.

The recent identification of an association between a variation (rs72665334) in intron 1 of *FGF14* and DBN of unknown aetiology (=“idiopathic”) in a genome-wide association study (GWAS)^10^ and the recurrent occurrence of DBN in GAA-*FGF14* disease^1^^,^^3^^,^^4^^,^^8^ suggest that *FGF14* intronic GAA expansions may represent a common genetic cause of DBN, which is the most common form of acquired persisting nystagmus yet remains undiagnosed in 30% of cases (idiopathic DBN).^11^^,^^12^

Here, we reassessed the molecular and clinical spectrum of GAA-*FGF14* disease by studying the frequency of *FGF14* (GAA)_≥250_ and (GAA)_200-249_ expansions in a large cohort of patients with idiopathic DBN and their phenotypic spectrum. We also retrospectively assessed the real-world and placebo-controlled treatment response of patients with GAA-*FGF14* disease to 4-AP.

## Methods

### Patient enrolment

**Fig. 1:**
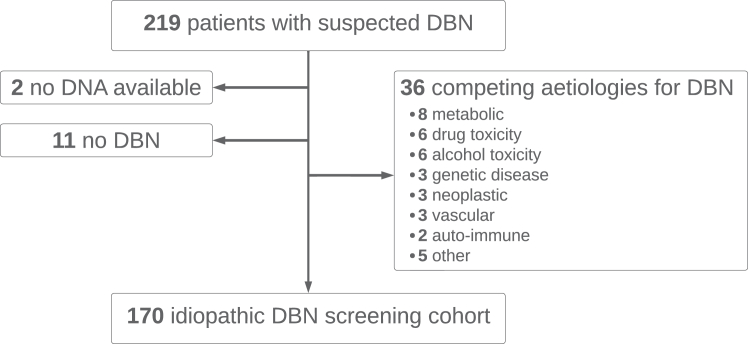
**Study flowchart of the recruitment of patients with idiopathic DBN**. DBN, downbeat nystagmus.

We retrospectively enrolled a consecutive series of 219 index patients with suspected DBN of unknown aetiology (idiopathic DBN) referred to the Department of Neurology or the German Center for Vertigo and Balance Disorders at the LMU Hospital in Munich, Germany, between 2012 and 2020. No formal sample size calculation was performed. Following referral, patients underwent a comprehensive aetiologic evaluation of DBN through a detailed medical history, assessment of drug abuse, comprehensive neurological and neuro-ophthalmological examination, laboratory tests, and brain imaging by MRI. Genetic screening for episodic and spinocerebellar ataxias was performed in select patients when deemed appropriate by the clinician. Patients were excluded from the study if: (i) no DNA was available for genetic screening (*n* = 2), (ii) DBN was not objectified on examination (*n* = 11), or (iii) a competing aetiology for DBN was identified (*n* = 36). The final study cohort comprised 170 patients with a diagnosis of idiopathic DBN (Fig. 1). Part of this patient cohort (*n* = 80) has previously been reported in the idiopathic DBN GWAS.^10^ All but three patients of Turkish descent were of self-reported European descent. Sex was self-reported by study participants, but was not further considered in statistical analyses.

### Deep phenotyping

Deep phenotyping was performed by systematically reassessing all medical records using a standardised data sheet while being blind to the GAA-*FGF14* genotype. Disease onset was defined as the age at onset of the first neurological symptoms related to the disease experienced by the patient. Functional impairment was assessed in terms of the need for mobility aids and by the Friedreich Ataxia Rating Scale (FARS) functional stage (0 = normal; 1 = minimal signs on examination; 2 = minimal disability; 3 = mild disability; 4 = moderate disability, requires a walker; 5 = severe disability, confined but can navigate a wheelchair; 6 = total disability).^13^ Results of routine brain MRI and nerve conduction studies were available for review in 70% (119/170) and 18% (31/170) of patients, respectively. Ancillary assessment of vestibular function was obtained in 94% (159/170) of patients using caloric stimulation (*n* = 147), video head impulse test (vHIT; *n* = 100), and rotatory chair test (*n* = 1). Posturography (Kistler platform) was obtained in 59% (100/170) of patients.^14^

Patients were stratified in one of four phenotypic clusters based on the presence of additional cerebellar and/or extracerebellar signs: pure DBN, DBN plus additional isolated cerebellar ocular motor signs (DBN + COM), DBN plus cerebellar ataxia (DBN + CA), and DBN plus cerebellar and extracerebellar features (DBN + EC), which included bilateral vestibulopathy (BVP) and/or polyneuropathy. Along with the cerebellar system, the vestibular and sensory systems appear to be preferentially impaired in GAA-*FGF14* disease.^3^^,^^8^ Additional cerebellar ocular motor signs were defined by the presence of at least one of: saccadic pursuit, dysmetric saccades, gaze-evoked nystagmus, rebound nystagmus, or impaired visual fixation suppression of the vestibulo-ocular reflex (VOR). Cerebellar ataxia was defined by the presence of cerebellar dysarthria, dysdiadochokinesia, intention tremor, ataxia of the upper limbs, or, when available, evidence of cerebellar involvement on posturography. BVP was diagnosed as per the consensus criteria of the Bárány Society requiring the documentation of bilaterally reduced or absent angular VOR function by caloric stimulation, vHIT, or rotatory chair.^15^ Polyneuropathy was diagnosed on nerve conduction studies (excluding focal entrapment neuropathies) or clinically defined by the combination of significantly decreased vibration sense at the ankles (≤3/8 on the Rydel–Seiffer scale) and decreased or absent ankle reflexes.^16^

### Assessment of treatment response to 4-aminopyridine

Assessment of treatment response to 4-AP was performed by two independent approaches. (1) *Open-label real-world treatment response data*. Information on clinician-reported and patient-reported response to 4-AP treatment (fampridine 10 mg twice a day) was collected (available for 83 patients). As all patients from this study who were treated with 4-AP as part of routine clinical care received the drug before the discovery of GAA-*FGF14* disease, the assessment of treatment response was naturally blind to the underlying GAA-*FGF14* genotype. Patients were routinely evaluated 1–3 months after initiating treatment with 4-AP. Patient-reported responses were assessed based on their global impressions of the impact of treatment on their neurological functioning, focusing on visual symptoms and/or gait unsteadiness, which are the two most commonly reported symptoms in patients with DBN.^12^ Clinician-reported responses were evaluated based on the clinician's impression of improvement of ocular motor signs (including DBN) and/or gait, which were previously suggested to be responsive to 4-AP in some patients with DBN.^17^ (2) *Double-blind placebo-controlled clinical trial data*. Four patients with DBN had been part of an earlier prospective placebo-controlled randomised double-blind trial assessing the efficacy of 4-AP in a cohort of 27 patients with DBN.^17^ All four patients, now known via the current study to carry an *FGF14* (GAA)_≥250_ expansion, were genetically undiagnosed at the time of the original trial.^17^ Due to unavailability of DNA, the genetic status of the remaining 23 patients who took part in the trial remains unknown. As part of the trial, patients had received 5 mg of 4-AP (or placebo) four times a day for 3 days and 10 mg of 4-AP (or placebo) four times a day for 4 days. Randomisation resulted in all four patients receiving active treatment first, then placebo, separated by a 1-week washout. Assessments were done before the first, 60 min after the first, and 60 min after the last drug administration, measuring the slow phase velocity (SPV) of DBN by video-oculography (degrees/second) as primary outcome.

### Genetic screening for *FGF14* repeat expansions

The *FGF14* repeat locus was genotyped as described previously using long-range PCR and bidirectional repeat-primed PCR.^18^ Expansions of at least 250 GAA-pure repeat units were considered pathogenic.^1^^,^^2^ Alleles of 200–249 GAA repeat units were analysed separately as their pathogenicity has recently been suggested.^19^

### Genotyping of the *FGF14* rs72665334 variant

The rs72665334 C > T variant in intron 1 of *FGF14* (GRCh38, chr13:102,150,076) was found to be associated with idiopathic DBN in 106 patients in a recent GWAS.^10^ Genotyping data were previously generated using the HumanOmniExpress-24 array and data for the rs72665334 variant with call rate probability >70% were extracted for 73 patients enrolled in this study to analyse whether this variant is in disequilibrium with the *FGF14* GAA repeat expansion.

We also genotyped the rs72665334 variant by Sanger sequencing in an independent and ethnically distinct cohort of 37 French-Canadian index patients with GAA-*FGF14* disease and DBN. PCR reactions were performed in a 24 μL volume using the Qiagen Taq DNA polymerase kit (catalog no. 201209, Qiagen) with 0.125 mM dNTPs, 1 μM of forward and reverse primers (forward primer: 5′-GCCCCTGTTCTAAAGCCTCT-3’; reverse primer: 5′-GATCGTCCAGCCACATCTCT-3′), and 240 ng of genomic DNA. Sanger sequencing of PCR amplification products was performed using the ABI 3730*xl* Analyzer (Applied Biosystems).

### Statistics

We assessed differences between groups with the non-parametric Mann–Whitney U test for continuous variables and the Fisher's exact test for categorical variables. The non-parametric Kruskal–Wallis test was used for comparisons between multiple groups. We calculated 95% confidence intervals (CIs) of frequency estimates using the adjusted Wald method to account for small groups. We calculated 95% CIs around positive predictive value estimates using the Wilson-Brown method. Effect sizes with 95% CIs are reported as risk ratio (RR) or odds ratio (OR) for tests of allelic association. Correlations were calculated using the Pearson's correlation coefficient for normally distributed variables or the Spearman's rank correlation coefficient for ordinal variables or non-normally distributed variables. The Kaplan–Meier method was used to analyse disease-free survival (R packages: survival and survminer). The Cox–Mantel test was used to compare survival until disease onset between groups. To estimate the longitudinal progression of disability over the disease course, we used a linear mixed-effects model of longitudinal FARS scores (considering the covariance between repeated scores of each subject) fitted by the restricted maximum likelihood method accounting for disease duration as fixed effect and with random intercepts and random slopes (R packages: lme4 and lmerTest).^20^ The 95% CI for the fixed effects estimate in the linear mixed-effects model was calculated using bootstrapping technique. We re-analysed treatment effects of 4-AP and placebo by means of a mixed-effect analysis of repeated measures, with post-hoc comparison by uncorrected Fisher's least significant difference test. We analysed the data in R (version 4.3) and GraphPad Prism 9.3. *p* value of <0.05 was considered significant, using the Benjamini-Hochberg method to correct for multiple comparisons. All analyses were two-sided.

### Ethics

This study was approved by the ethics committees of the LMU Munich (#379-11), the Montreal Neurological Hospital-Institute (#MPE-CUSM-15-915), and Clinical Trials Ontario (#1577 CTO). We obtained written informed consent from all the participants in this study. We re-analysed the video-oculography data from four patients recruited in Munich, Germany who had given written informed consent to participate in an earlier randomised placebo-controlled double-blind trial of 4-AP for DBN approved by the ethics committee of the LMU Munich (#285-04).^17^

### Data availability

Individual deidentified patient data may be shared at the request of any qualified investigator upon reasonable request. No consent for open sharing has been obtained.

### Role of funders

The funders had no role in study design, data collection, analysis, interpretation of data, writing of the report, and in the decision to submit the paper for publication.

## Results

### Frequency of *FGF14* GAA repeat expansions in downbeat nystagmus syndromes

**Fig. 2:**
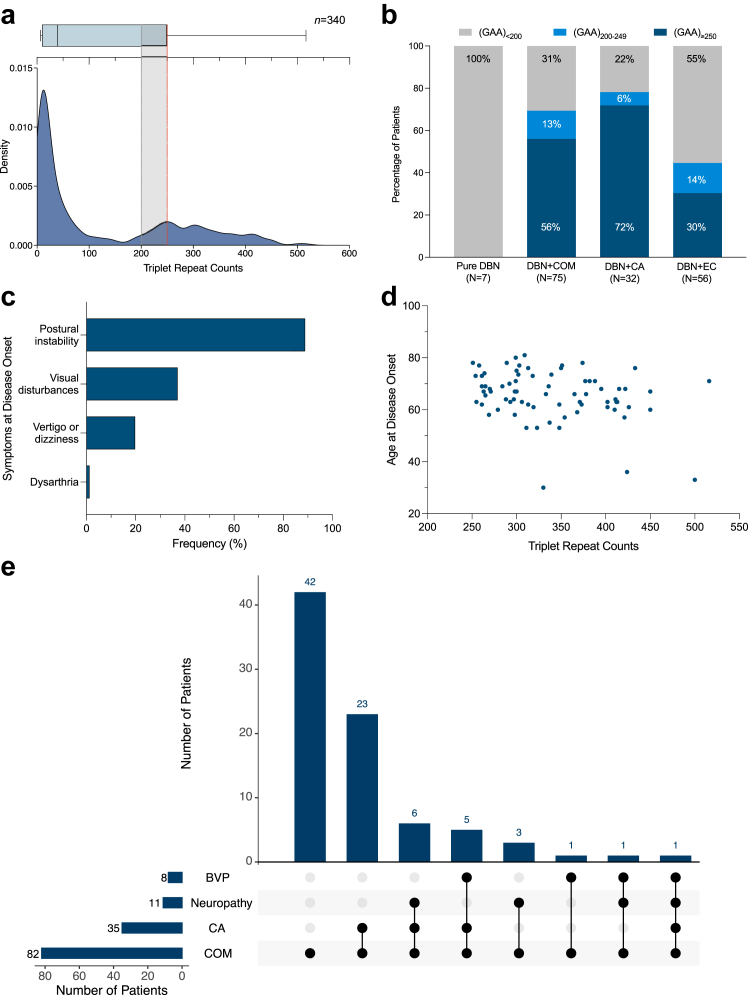
**Frequency of *FGF14* GAA repeat expansions in DBN syndromes**. (a) Allele distribution of the *FGF14* repeat locus in 170 patients with idiopathic DBN syndromes (340 chromosomes). The density plot shows allele size frequencies, with higher densities indicating greater frequencies. The box-and-whisker plot above the graph shows the allele distribution. The box indicates the 25th percentile (first quartile), the median, and the 75th percentile (third quartile), and the whiskers indicate the 25th percentile minus 1.5 x IQR and the 75th percentile plus 1.5 x IQR. Outliers are represented by black dots. The dashed gray line and the shaded gray area indicate the so-called “intermediate” allele range of (GAA)_200-249_, and the dashed red line represents the pathogenic threshold of (GAA)_≥250_ repeat units. (**b**) Percentage of patients carrying an *FGF14* (GAA)_≥250_ expansion (dark blue), an *FGF14* (GAA)_200-249_ allele (light blue), and an *FGF14* (GAA)_<200_ allele (gray) in the subgroups with (1) pure DBN (0/7, 0/7, and 7/7 patients, respectively), (2) DBN plus additional isolated cerebellar ocular motor signs (DBN + COM) (42/75, 10/75, and 23/75 patients), (3) DBN plus cerebellar ataxia (DBN + CA) (23/32, 2/32, and 7/32 patients), and (4) DBN plus cerebellar ocular motor signs and/or ataxia and extracerebellar features (DBN + EC) (17/56, 8/56, and 31/56 patients). (**c**) Frequency of presenting symptoms in 81 patients with DBN carrying an *FGF14* (GAA)_≥250_ expansion. Data on presenting symptoms were missing for one patient. Patients may present with multiple symptoms at disease onset. Visual disturbances include diplopia, oscillopsia, and visual blurring. (**d**) Weak inverse association between size of the repeat expansion and age at disease onset in 74 patients carrying an *FGF14* (GAA)_≥250_ expansion for whom information on age at onset was available (Spearman's r, −0.27; 95% CI, −0.47 to −0.03; *p* = 0.022). (**e**) UpSet plot showing co-occurrence of cerebellar ocular motor signs (COM), cerebellar ataxia (CA), bilateral vestibulopathy (BVP), and polyneuropathy among 82 patients with DBN carrying an *FGF14* (GAA)_≥250_ expansion.

A total of 170 index patients with idiopathic DBN were screened for *FGF14* GAA repeat expansions (Figs. 1 and 2a). We identified 82 patients (48%) who carried an *FGF14* (GAA)_≥250_ expansion (median size, 324 repeat units; interquartile range [IQR], 289–388), including one patient carrying biallelic GAA expansions of 280 and 313 repeat units. Alleles of 200–249 repeat units were identified in 12% of patients (20/170; median size, 234 repeat units; IQR, 225–243), compared to 0.87% in 2191 previously reported control individuals^21^ (19/2191), suggesting an enrichment of this population of alleles in patients with DBN (OR, 15.20; 95% CI, 7.52–30.80; Fisher's exact test, *p* < 0.0001).

The frequency of *FGF14* (GAA)_≥250_ expansions in DBN syndromes stratified by phenotype was 56% (42/75) for DBN + COM, 72% (23/32) for DBN + CA, and 30% (17/56) for DBN + EC. None of the seven patients with pure DBN (median disease duration, 3 years; IQR, 1–10) was found to carry an *FGF14* (GAA)_≥250_ repeat expansion (Fig. 2b).

### Phenotype and discriminative features

Table 1 summarises the baseline characteristics and clinical features of patients carrying a (GAA)_≥250_ expansion, a (GAA)_200–249_ allele, and a (GAA)_<200_ allele.

**Table 1:** Characteristics and discriminative features of the DBN endophenotypic cluster of GAA-*FGF14* disease.

	*FGF14* (GAA)_≥250_ (*n* = 82)	*FGF14* (GAA)_200-249_ (*n* = 20)	*FGF14* (GAA)_<200_ (*n* = 68)	*FGF14* (GAA)_≥250_ vs *FGF14* (GAA)_<200_	
				*p* value	*q* value
Male sex	44 (54%)	11 (55%)	40 (59%)	–	–
Female sex	38 (46%)	9 (45%)	28 (41%)	–	–
Triplet repeat count of the larger allele^a^	324 (289–388)	234 (225–243)	37 (15–78)	–	–
Age at disease onset	67 (61.75–71.5)	72 (64–77.5)	66 (54.75-75)	0.61	0.76
Disease duration	6 (3–8.25)	4 (2–9)	4 (2–8)	0.17	0.41
Age at last examination	73 (68–78)	79 (73–82.75)	72 (61–78)	0.34	0.54
Positive family history	31/82 (38%)	4/20 (20%)	13/67 (19%)	0.019	0.097
FARS disability stage^b^	3 (2–3)	3 (2–3)	2.5 (2–3)	0.19	0.41
History of falls	27/50 (54%)	8/14 (57%)	20/35 (57%)	0.83	0.88
Regular use of walking aid	16/81 (20%)	4/19 (21%)	15/66 (23%)	0.69	0.76
***Symptoms***					
Episodic symptoms	11/81 (14%)	0/19 (0%)	18/66 (27%)	0.059	0.17
Gait impairment	81/81 (100%)	20/20 (100%)	68/68 (100%)	1.00	1.00
Vertigo or dizziness	23/82 (28%)	10/20 (50%)	26/68 (38%)	0.22	0.44
Visual disturbances	47/82 (57%)	8/20 (40%)	30/68 (44%)	0.14	0.37
Fine motor impairment	12/80 (15%)	1/18 (6%)	12/66 (18%)	0.66	0.76
Speech impairment	13/81 (16%)	4/19 (21%)	13/66 (20%)	0.66	0.76
***Clinical signs***					
Impaired balance/gait	67/79 (85%)	13/19 (68%)	44/64 (69%)	0.027	0.11
Cerebellar ocular motor signs					
Gaze-evoked nystagmus	57/82 (70%)	11/20 (55%)	42/67 (63%)	0.39	0.59
Saccadic pursuit	80/81 (99%)	20/20 (100%)	59/67 (88%)	0.011	0.073
Dysmetric saccades	22/79 (28%)	6/20 (30%)	22/67 (33%)	0.59	0.76
Impaired VOR suppression^c^	64/74 (86%)	16/17 (94%)	23/46 (50%)	<0.0001	0.00067
Cerebellar ataxia					
Ataxia of upper limbs	16/73 (22%)	1/14 (7%)	21/55 (38%)	0.051	0.16
Dysdiadochokinesia	15/71 (21%)	1/16 (6%)	14/54 (26%)	0.53	0.76
Dysarthria	12/80 (15%)	2/19 (11%)	12/63 (19%)	0.65	0.76
Tremor of upper limbs	12/79 (15%)	2/18 (11%)	6/66 (9%)	0.32	0.54
Neuropathy					
Impaired vibration at ankle (≤3/8)	11/79 (14%)	6/18 (33%)	24/65 (37%)	0.0017	0.018
Ankle hyporeflexia	20/79 (25%)	4/18 (22%)	29/65 (45%)	0.021	0.097
Pyramidal tract signs	1/77 (1%)	0/18 (0%)	2/66 (3%)	0.59	0.76
***MRI***					
Vermis atrophy	8/60 (13%)	1/11 (9%)	10/47 (21%)	0.31	0.54
Cerebellar hemisphere atrophy	5/60 (8%)	0/11 (0%)	8/47 (17%)	0.23	0.44
Supratentorial atrophy	5/60 (8%)	2/10 (20%)	4/47 (9%)	1.00	1.00
Brainstem atrophy	0/60 (0%)	0/10 (0%)	2/47 (4%)	0.19	0.41
***Nerve conduction studies***					
Axonal neuropathy	7/15 (47%)	3/5 (60%)	10/11 (91%)	0.036	0.13
***Vestibular function evaluation****—caloric stimulation, vHIT, rotatory chair*					
Bilateral vestibulopathy	8/79 (10%)	3/18 (17%)	21/62 (34%)	0.0007	0.011
VOR gain on vHIT in BVP	0.44 (0.30–0.53)	0.21 (0.06–0.35)	0.11 (0.03–0.30)	0.0062	0.049

Data are reported as frequencies (percentages) for qualitative variables and median (IQR) for quantitative variables. Data on age at onset were missing for eight patients in the (GAA)_≥250_ group, three patients in the (GAA)_200-249_ group, and 10 patients in the (GAA)_<200_ group.

Abbreviations: BVP, bilateral vestibulopathy; FARS, Friedreich Ataxia Rating Scale; vHIT, video head impulse test; VOR, vestibulo-ocular reflex.

a The *FGF14* (GAA)_≥250_ group includes a patient carrying biallelic GAA repeat expansions (280 and 313 repeat units). The *FGF14* (GAA)_<200_ group includes two patients carrying a likely non-pathogenic (GAAGGA)_n_ expansion^19^^,^^46^ (335 and 319 triplet repeat units equivalent, respectively).

b Last available FARS disability stage measured off 4-aminopyridine.

c In patients without bilateral vestibulopathy.

Symptoms in patients carrying an *FGF14* (GAA)_≥250_ repeat expansion started at a median age of 67 years (IQR, 61.75–71.50). The most common presenting symptom was gait unsteadiness (89%; 72/81), followed by visual disturbances (37%; 30/81), such as diplopia, oscillopsia, or visual blurring, vertigo and/or dizziness (20%; 16/81), and, rarely, dysarthria (1%; 1/81) (Fig. 2c). We observed a weak inverse association between the age at onset and the size of the repeat expansion (Spearman's r, −0.27; 95% CI, −0.47 to −0.03; *p* = 0.022) (Fig. 2d). Episodic symptoms consisting in gait and limb ataxia, vertigo, and visual disturbances were experienced by 14% of patients (11/81) and first manifested at a median age of 61.5 years (IQR, 48.75–67.75). All patients with episodic symptoms exhibited interictal DBN. Family history for DBN or ataxia was positive in 31 of 82 patients (38%), of whom 26 had evidence of autosomal dominant inheritance. In keeping with the likely reduced male transmission of GAA-*FGF14* disease, we observed that only 2 of 26 dominantly inherited cases (8%) were paternally inherited. There were no significant differences in clinical and ancillary findings between patients with and without positive family history.

The median age and disease duration at last examination were 73 years (IQR, 68–78) and 6 years (IQR, 3–8.25), respectively. Consistent with a recent case series of two patients with GAA-*FGF14* disease,^22^ patients carrying a (GAA)_≥250_ expansion from our study exhibited spontaneous DBN in primary position or DBN evoked with horizontal and vertical gaze. Patients displayed DBN even without changes in head position. Additional cerebellar ocular motor signs were observed in 100% (82/82), cerebellar ataxia in 43% (35/82), and extracerebellar features in 21% (17/82) of patients carrying a (GAA)_≥250_ expansion (Fig. 2e). Cerebellar ocular motor signs comprised saccadic pursuit (99%; 80/81), impaired visual fixation suppression of the VOR (86%; 64/74 in patients without BVP), gaze-evoked nystagmus (70%; 57/82), dysmetric saccades (28%; 22/79), and rebound nystagmus (20%; 16/82). Signs of cerebellar ataxia included dysmetria of upper limbs (46%; 16/35), dysdiadochokinesia (47%; 15/32), dysarthria (35%; 12/34), and intention tremor (11%; 4/35). Cerebellar involvement reflected by ∼3Hz titubation was identified in 18 of 55 patients (33%) on posturography. Brain MRI of eight patients showed cerebellar atrophy (13%; 8/60), which was limited to the vermis in three patients and extended to the hemispheres in five patients.

The subgroup of patients carrying a (GAA)_≥250_ expansion with DBN plus cerebellar ocular motor signs and/or ataxia and extracerebellar features comprised nine patients with polyneuropathy, six patients with BVP, and two patients with polyneuropathy plus BVP (Fig. 2e). Polyneuropathy was diagnosed on nerve conduction studies in seven patients and clinically in four patients. Results of nerve conduction studies were consistent with length-dependent sensorimotor axonal polyneuropathy in six patients and sensory polyneuropathy in one patient. BVP was diagnosed in 8 of 79 patients (10%) by caloric stimulation (*n* = 1) and vHIT (*n* = 7).

To identify discriminative features of the DBN endophenotypic cluster of GAA-*FGF14* disease, we compared the phenotypic features of patients carrying a (GAA)_≥250_ expansions to that of patients carrying a (GAA)_<200_ allele (Table 1). Both groups did not differ significantly in terms of disease duration, baseline characteristics, and functional impairment. Impairment of the visual fixation suppression of the VOR was significantly more common in patients carrying a (GAA)_≥250_ expansions (86%, 64/74 vs 50%, 23/46; RR, 1.73; 95% CI, 1.28–2.34; Fisher's exact test, *p* < 0.0001, *q* = 0.00067). Impaired vibration at the ankles (14%, 11/79 vs 37%, 24/65; RR, 0.38; 95% CI, 0.20–0.71; Fisher's exact test, *p* = 0.0017, *q* = 0.018) and BVP (10%, 8/79 vs 34%, 21/62; RR, 0.30; 95% CI, 0.14–0.63; Fisher's exact test, *p* = 0.0007, *q* = 0.011) were significantly less common in patients carrying a (GAA)_≥250_ expansion than in patients carrying a (GAA)_<200_ allele. In the subset of patients in whom BVP was documented by vHIT, the mean VOR gain was significantly higher (values of >0.6 are considered normal) in patients carrying a (GAA)_≥250_ expansion compared to patients carrying a (GAA)_<200_ allele (0.44 [IQR, 0.30–0.53] vs 0.11 [IQR, 0.03–0.30]; Mann–Whitney U test, *p* = 0.0062, *q* = 0.049), despite no significant difference in disease duration between the groups (median, 14.5 vs 8 years; Mann–Whitney U test, *p* = 0.15).

### Disease progression

**Fig. 3:**
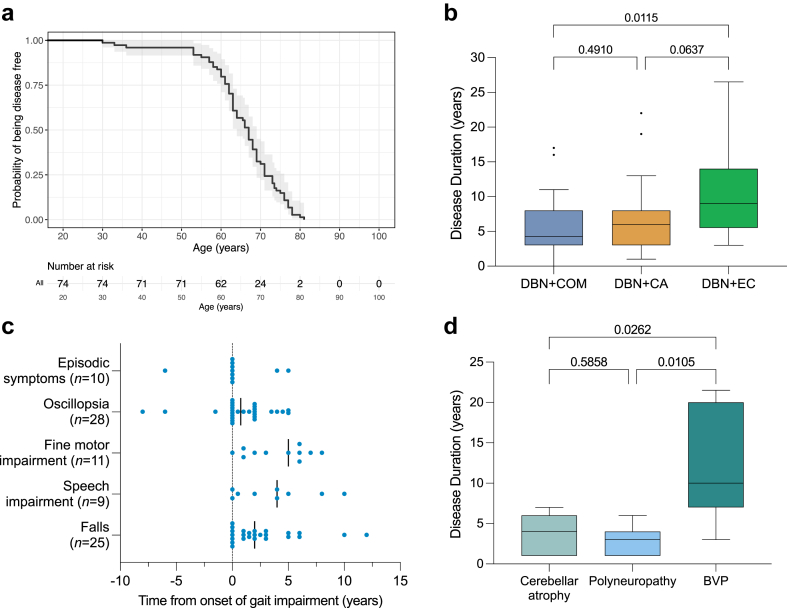
**Phenotypic evolution of the DBN endophenotypic cluster of GAA-*FGF14* disease**. (a) Kaplan–Meier curve showing the probability of being disease free as a function of the age (years) for 74 patients carrying an *FGF14* (GAA)_≥250_ expansion for whom age at onset was available. Median survival before occurrence of disease was 67 years. For clarity, ages below 20 years were not included on the graph, as none of the patients developed GAA-*FGF14* disease before the age of 30 years. The analysis commences upon the onset of GAA-*FGF14* disease in the first patient and ends when all patients in the cohort have developed the disease. The shaded gray area displays the 95% confidence interval around the probability estimate. (**b**) Disease duration at last examination for patients carrying an *FGF14* (GAA)_≥250_ expansion with DBN plus additional isolated cerebellar ocular motor signs (DBN + COM, *n* = 36; median, 4.25 years; IQR, 3–8 years), DBN plus cerebellar ataxia (DBN + CA, *n* = 21; median, 6 years; IQR, 3–8 years), and DBN plus cerebellar ocular motor signs and/or ataxia and extracerebellar features (DBN + EC, *n* = 17; median, 9 years; IQR, 5.5–14). Disease duration was significantly longer for patients with extracerebellar features (Kruskal–Wallis test, *p* = 0.014). (**c**) Temporal evolution of select phenotypic features relative to the onset of gait impairment (dotted line). The solid black lines show the median time from onset of gait impairment for each individual feature. (**d**) Occurrence of cerebellar atrophy on brain MRI (*n* = 7), polyneuropathy on nerve conduction studies (*n* = 7), and BVP on caloric stimulation or vHIT (*n* = 7) in relation to disease onset. BVP occurred later in disease course compared to cerebellar ataxia and polyneuropathy (Kruskal–Wallis test, *p* = 0.0041). In panels b and d, the box indicates the 25th percentile (first quartile), the median, and the 75th percentile (third quartile), and the whiskers indicate the 25th percentile minus 1.5 x IQR and the 75th percentile plus 1.5 x IQR. Outliers are represented by black dots. In panels b and d, the individual between-group adjusted *p* values (*q* values) as per the Benjamini-Hochberg method are shown in the graphs. Adjusted *p* values < 0.05 indicate statistically significant difference.

We used a Kaplan–Meier survival analysis to model the age at disease onset of patients carrying a (GAA)_≥250_ expansion, estimating their probability of remaining disease-free over time (Fig. 3a). Survival until disease onset in patients carrying a (GAA)_≥250_ expansion did not significantly differ between the three main phenotypic subgroups (median age at onset, 67 years [IQR, 61–72.5] for DBN + COM vs 67 years [IQR, 62.5–72] for DBN + CA vs 66 years [IQR, 63–72] for DBN + EC; Cox–Mantel test, *p* = 0.94). However, disease duration at last examination was significantly longer in patients with DBN plus cerebellar and extracerebellar features (median, 4.25 years [IQR, 3–8] for DBN + COM vs 6 years [IQR, 3–8] for DBN + CA vs 9 years [IQR, 5.5–14] for DBN + EC; Kruskal–Wallis test, *p* = 0.014) (Fig. 3b), suggesting that extracerebellar features may develop later in the disease course.

We also studied the evolution of key symptoms in patients carrying a (GAA)_≥250_ expansion in relation to gait impairment (Fig. 3c). Episodic symptoms (median, 0 year; IQR, 0–1) and oscillopsia (0.75 year; IQR, 0–2) manifested concurrently with gait impairment in most patients. However, episodic symptoms and oscillopsia preceded gait impairment in 1 of 10 (10%) and 3 of 28 (11%) patients for whom information on age at onset of these features was available, respectively. In comparison, fine motor impairment (5 years; IQR, 1–6), speech impairment (4 years; IQR, 0.25–6.5), and falls (2 years; 0–4) developed later in the disease course. Sixteen patients (20%; 16/81) eventually required a walking aid after a median disease duration of 4 years (IQR, 2–11), including three patients (4%; 3/81) who became wheelchair dependent after a median disease duration of 8 years (IQR, 7–12). Further to this analysis, we observed that BVP developed at a significantly later stage in the disease compared to cerebellar atrophy and polyneuropathy (median disease duration at first occurrence, 4 years [IQR, 1–6] for cerebellar atrophy vs 3 years [IQR, 1–4] for polyneuropathy vs 10 years [IQR, 7–20] for BVP; Kruskal–Wallis test, *p* = 0.0041) (Fig. 3d).

**Fig. 4:**
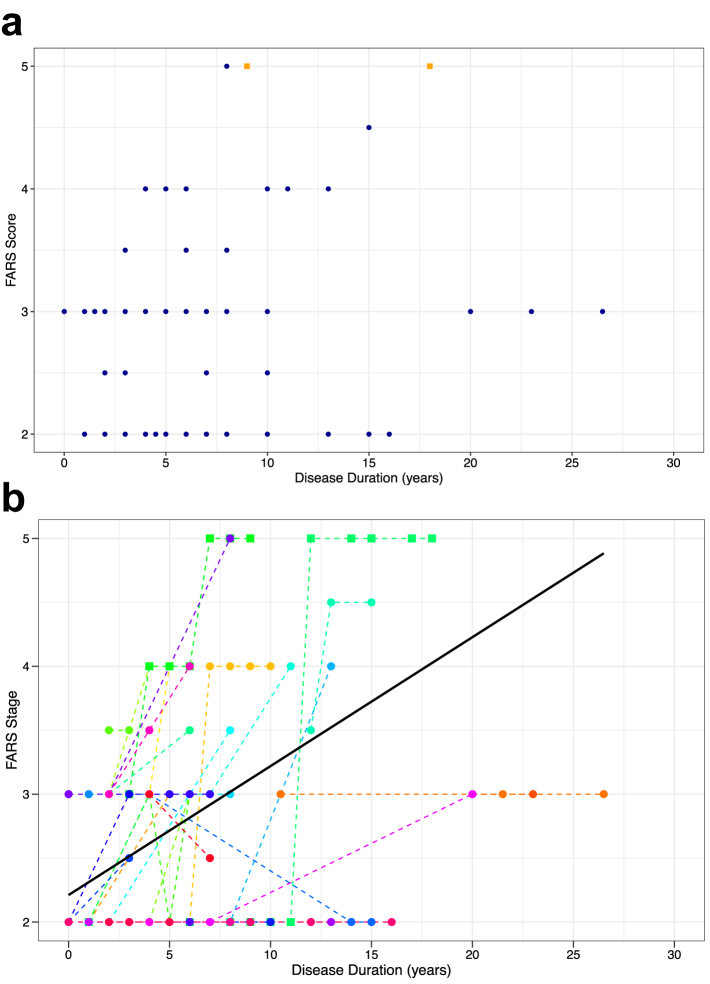
**Progression of disability of the DBN endophenotypic cluster of GAA-*FGF14* disease**. (a) Cross-sectional progression of the functional impairment as assessed by the FARS functional disability stage relative to disease duration (*n* = 73 patients carrying an *FGF14* [GAA]_≥250_ expansion). The FARS functional stage was not significantly associated with disease duration (Spearman's r, 0.194; 95% CI, −0.04–0.41; *p* = 0.10). (**b**) Longitudinal intra-individual progression of functional impairment as assessed by the FARS functional disability stage relative to disease duration (148 observations from 40 patients carrying an *FGF14* [GAA]_≥250_ expansion are shown). Observations from the same patient are connected by a dotted line. The solid black line shows the average progression of the FARS stage over disease duration across all patients as modelled by a linear mixed-effects model accounting for disease duration as fixed effect and with random intercepts and random slopes. In both panels, only FARS stages measured off 4-AP treatment are shown. Two patients who became wheelchair-dependent following a hip fracture and a prolonged medical admission, respectively, are represented by orange squares in panel a and by squares in panel b. These patients also carried alleles of 218 and 270 repeat units and 280 and 313 repeat units, respectively.

At time of last examination, the median FARS functional stage measured off 4-AP was 3 (IQR, 2–3), indicating mild disability. We found no statistically significant association between the FARS stage and disease duration (Spearman's r, 0.194; 95% CI, −0.04 to 0.41; *p* = 0.10) (Fig. 4a). Longitudinal data in 40 patients (148 observations) showed an overall slow intra-individual increase of 0.10 FARS stage per year of disease (95% CI, 0.05–0.15; t-test using Satterthwaite's method for approximating degrees of freedom, *p* = 0.00012) (Fig. 4b). While disease evolved relatively slowly in most patients, inspection of individual disability progression trajectories revealed inter-individual variability in rates of progression (Fig. 4b). Concurrent medical conditions substantially contributed to disability burden in two patients. One patient became wheelchair-bound following a prolonged medical admission for paralytic ileus. However, this patient also carried biallelic expansions (280 and 313 repeat units) which may have contributed to the faster disability progression. The second patient, carrying alleles of 218 and 270 repeat units, became wheelchair-bound following a hip fracture.

### Patients carrying a (GAA)_200-249_ allele have a similar phenotype as patients carrying a (GAA)_≥250_ allele

While previous studies have established a preliminary pathogenic threshold of ≥250 GAA repeat units,^1^^,^^2^ the pathogenic potential of (GAA)_200–249_ alleles has recently been suggested following the identification of a family with late-onset slowly progressive ataxia in which (GAA)_200-249_ and (GAA)_≥250_ alleles segregated with disease.^19^ As previously mentioned, alleles of 200–249 GAA repeat units were significantly enriched in patients with DBN from our study compared to controls (12%, 20/170 vs 0.87%, 19/2191; OR, 15.20; 95% CI, 7.52–30.80; Fisher's exact test, *p* < 0.0001), thus providing further support for their potential pathogenicity.

We next aimed to characterise the phenotype of patients carrying a (GAA)_200-249_ allele to assess whether it was similar to that of patients carrying a (GAA)_≥250_ allele (Table 1). Patients carrying a (GAA)_200-249_ allele did not significantly differ from patients carrying a (GAA)_≥250_ allele in terms of baseline characteristics, clinical features, and progression of functional disability. In comparison, impairment of the visual fixation suppression of the VOR, which was strongly associated with *FGF14* (GAA)_≥250_ expansions, was significantly more common in patients carrying a (GAA)_200-249_ allele compared to patients carrying a (GAA)_<200_ allele (94% [95% CI, 71–100%], 16/17 vs 50% [95% CI, 36–64%], 23/46; RR, 1.88; 95% CI, 1.38–2.57; Fisher's exact test, *p* = 0.0012; *q* = 0.037), but not significantly different from patients carrying a (GAA)_≥250_ allele (94% [95% CI, 71–100%], 16/17 vs 86% [95% CI, 77–93%], 64/74; Fisher's exact test, *p* = 0.68; *q* = 1.00).

### Association of the rs72665334 variant with *FGF14* (GAA)_≥250_ expansions

**Fig. 5:**
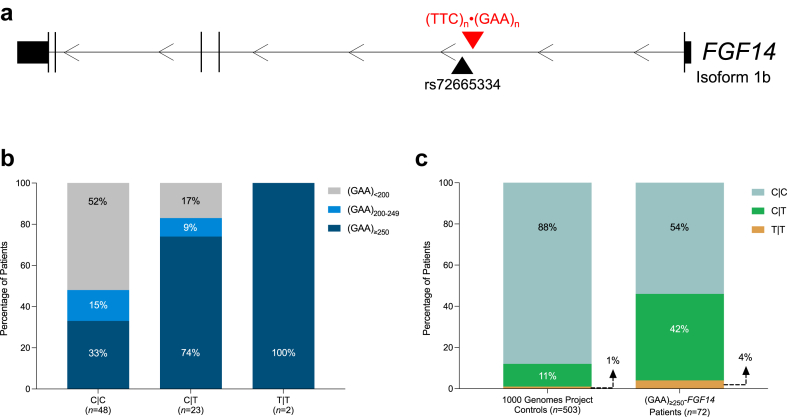
**Association of the rs72665334 variant with *FGF14* GAA expansions**. (a) Diagram of the *FGF14* gene, isoform 1b showing the location of the rs72665334 variant (GRCh38, chr13:102,150,076) in relation to the (GAA)·(TTC) repeat locus in the first intron (GRCh38, chr13:102,161,575-102,161,726). The rs72665334 variant is located ∼11 kb 5′ of the intronic GAA short tandem repeat. (**b**) Percentage of patients with DBN carrying an *FGF14* (GAA)_≥250_ expansion (*n* = 35; dark blue), an *FGF14* (GAA)_200-249_ allele (*n* = 9; light blue), and an *FGF14* (GAA)_<200_ allele (*n* = 29; gray) with the C|C, C|T, and T|T rs72665334 genotypes. There was a significantly greater proportion of patients with a C|T or T|T genotype who carried an *FGF14* (GAA)_≥250_ expansion compared to an *FGF14* (GAA)_<200_ allele (54%, 19/35 vs 14%, 4/29; OR, 7.18; 95% CI, 1.91–34.43; Fisher's exact test *p* = 0.0014). (**c**) Frequency of the C|C, C|T, and T|T rs72665334 genotypes in 503 European controls from the 1000 Genomes Project^23^ and 72 patients with DBN carrying an *FGF14* (GAA)_≥250_ expansion (*n* = 35 from the idiopathic DBN cohort and *n* = 37 from the French-Canadian cohort). The C|T and T|T genotypes were significantly more frequent in patients with DBN carrying an *FGF14* (GAA)_≥250_ expansion compared to controls (46%, 33/72 vs 12%, 60/503; OR, 6.22; 95% CI, 3.51–11.02; Fisher's exact test, *p* < 0.0001).

A recent GWAS has identified an association between idiopathic DBN and the *FGF14* rs72665334 C > T variant in 106 patients (Fig. 5a).^10^ This same variant was also previously found to be part of a disease haplotype shared by three Australian patients with GAA-*FGF14* disease in a previous study.^2^ Given the frequent occurrence of DBN in GAA-*FGF14* disease, we therefore hypothesised that the rs72665334 variant might be in disequilibrium with the *FGF14* GAA repeat expansion, which is located ∼11 kb away (Fig. 5a). In our cohort, we observed that a significantly greater proportion of patients with a C|T or T|T genotype carried an *FGF14* (GAA)_≥250_ expansion compared to an *FGF14* (GAA)_<200_ allele (54%, 19/35 vs 14%, 4/29; OR, 7.18; 95% CI, 1.91–34.43; Fisher's exact test, *p* = 0.0014) (Fig. 5b). Similarly, we also found that 38% of patients (14/37) from an independent and ethnically distinct cohort of 37 French-Canadian index patients with GAA-*FGF14* disease and DBN had a C|T or T|T rs72665334 genotype. To assesses whether the rs72665334 variant is in disequilibrium with the *FGF14* (GAA)_≥250_ expansion at a population level, we compared the frequency of the C|T and T|T genotypes in 503 European controls from the 1000 Genomes Project^23^ and 72 patients with GAA-*FGF14* disease and DBN (35 patients from this study and 37 French-Canadian patients). The C|T and T|T genotypes were significantly more frequent in patients carrying an *FGF14* (GAA)_≥250_ expansion compared to controls (46%, 33/72 vs 12%, 60/503; OR, 6.22; 95% CI, 3.51–11.02; Fisher's exact test, *p* < 0.0001) (Fig. 5c). Together, these results show that the rs72665334 variant is in disequilibrium with the *FGF14* GAA repeat expansion. However, its absence in 54% of patients carrying an *FGF14* (GAA)_≥250_ expansion suggests that *FGF14* GAA repeat expansions may arise on distinct haplotype backgrounds.

### Treatment response to 4-aminopyridine

#### Analysis of open-label real-world treatment response data

**Fig. 6:**
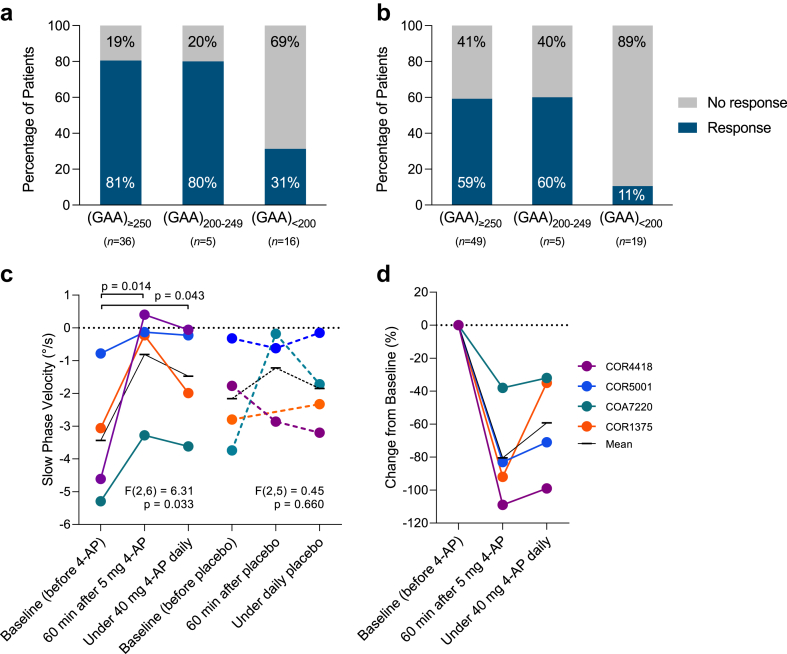
**Treatment response to 4-aminopyridine**. *Open-label real-world treatment response data*. Percentage of patients presenting (**a**) a clinician-reported response and (**b**) a patient-reported response to 4-AP treatment in the subgroups with an *FGF14* (GAA)_≥250_ expansion (29/36 and 29/49 patients, respectively), (GAA)_200-249_ allele (4/5 and 3/5 patients), and (GAA)_<200_ allele (5/16 and 2/19 patients). *Double-blind placebo-controlled clinical trial data*. (**c**) Effect of 4-AP on the slow phase velocity (SPV) of DBN in a randomised double-blind trial.^17^ Compared to baseline, the absolute value of the SPV significantly decreased under 4-AP 60 min after the first dose and under daily treatment, but not under placebo. Note that all four patients were randomised to receive active treatment first (=4-AP) and placebo second. SPV during all three measurements of the placebo phase was higher than at the baseline of the treatment phase. The improved values of the SPV under 4-AP were in the range of the placebo values. (**d**) Treatment effect of 4-AP, illustrated by relative reduction (improvement) of SPV as potential future trial outcome assessment. The genotype of the four patients was: COR4418, 65 and 298 repeat units; COR5001, 44 and 354 repeat units; COA7220, 16 and 265 repeat units; COR1375, 280 and 313 repeat units. In panels c and d, the black line shows the mean value for all four patients at each time point.

**Fig. 7:**
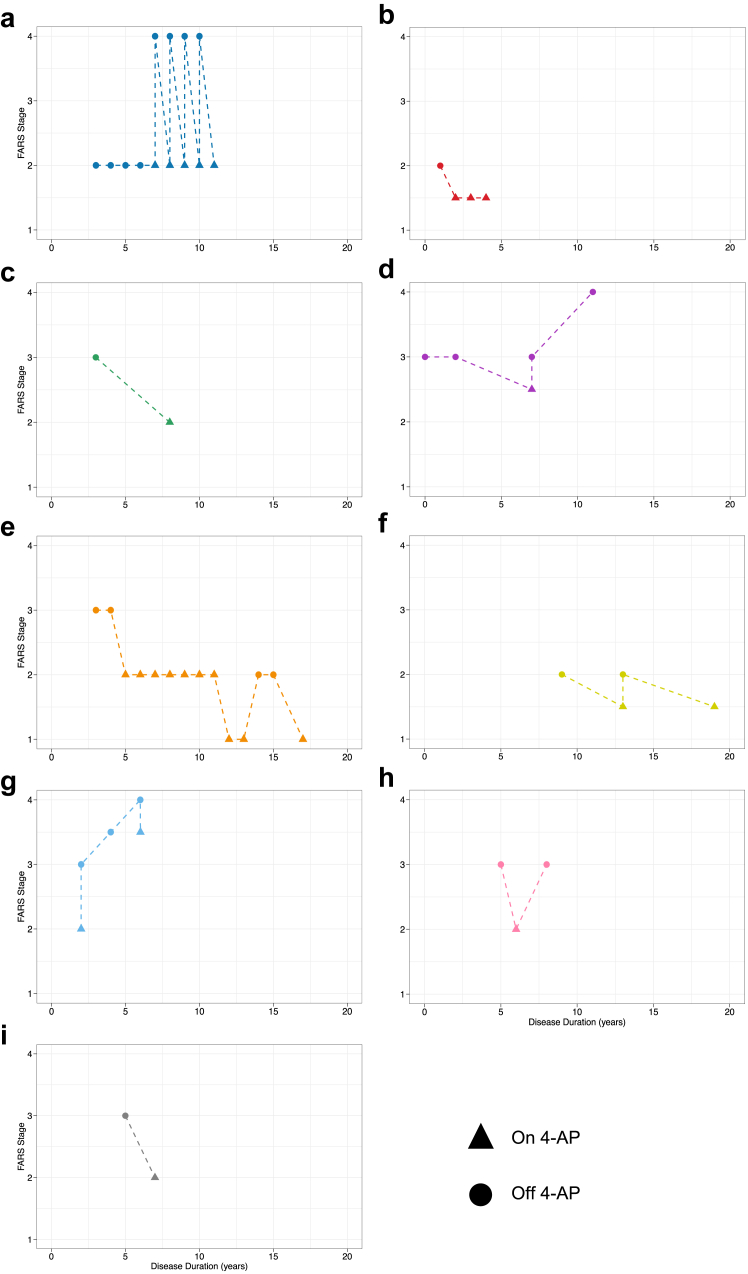
**Effect of 4-aminopyridine on functional impairment**. Longitudinal intra-individual progression of functional impairment while on and off 4-AP treatment as assessed by the FARS functional disability stage relative to disease duration for (**a–g**) seven patients carrying an *FGF14* (GAA)_≥250_ expansion and (**h and i**) two patients carrying an *FGF14* (GAA)_200-249_ allele. FARS stages were lower while on 4-AP treatment for all nine patients, indicating an improvement in the level of disability. No patients carrying an *FGF14* (GAA)_<200_ allele had FARS stages recorded while on and off 4-AP treatment. The genotype of the nine patients was: (**a**) 29 and 263 repeat units; (**b**) 18 and 292 repeat units; (**c**) 9 and 348 repeat units; (**d**) 16 and 288 repeat units; (**e**) 8 and 500 repeat units; (**f**) 16 and 424 repeat units; (**g**) 16 and 336 repeat units; (**h**) 34 and 223 repeat units; (**i**) 9 and 242 repeat units.

We assessed the response to 4-AP treatment in patients with DBN stratified by *FGF14* genotype (open-label treatment as part of routine clinical care). Treatment response for all patients had been recorded before the discovery of GAA-*FGF14* disease and, therefore, both clinicians and patients were naturally blind to the underlying GAA-*FGF14* genotype. A clinician-reported treatment response was recorded for 29 of 36 patients carrying a (GAA)_≥250_ allele (81%; improvement of ocular motor signs, including DBN: 24 patients; and gait: 14 patients), 4 of 5 patients carrying a (GAA)_200-249_ allele (80%; improvement of ocular motor signs, including DBN: 2 patients; and gait: 3 patients), and 5 of 16 patients carrying a (GAA)_<200_ allele (31%; improvement of ocular motor signs, including DBN: 3 patients; and gait: 2 patients) (Fig. 6a). A patient-reported benefit was recorded for 29 of 49 patients carrying a (GAA)_≥250_ allele (59%; improvement of visual symptoms: 14 patients; and gait: 17 patients), 3 of 5 patients carrying a (GAA)_200-249_ allele (60%; improvement of visual symptoms: 2 patients; and gait: 2 patients), and 2 of 19 patients carrying a (GAA)_<200_ allele (11%; improvement of visual symptoms: 1 patient; and gait: 1 patient) (Fig. 6b). Of note, one patient carrying a (GAA)_≥250_ allele had a complete cessation of episodes of ataxia with 4-AP. Patients carrying a (GAA)_≥250_ or a (GAA)_200-249_ allele had a significantly greater clinician-reported (80%, 33/41 vs 31%, 5/16; RR, 2.58; 95% CI, 1.23–5.41; Fisher's exact test *p* = 0.0011) and patient-reported (59%, 32/54 vs 11%, 2/19; RR, 5.63; 95% CI, 1.49–21.27; Fisher's exact test *p* = 0.00033) response rate to 4-AP treatment compared to patients carrying a (GAA)_<200_ allele. To next evaluate the effect of 4-AP on disability, we examined the FARS disability stages assessed on and off 4-AP. Data were available for seven patients carrying a (GAA)_≥250_ allele and two patients carrying a (GAA)_200-249_ allele (Fig. 7). Remarkably, the FARS stages for all nine patients were lower, by up to 2 stages, while on 4-AP compared to off 4-AP (Fig. 7). The response to 4-AP, whether reported by the clinician or the patient, may also be a useful clinical sign to increase suspicion for GAA-*FGF14* disease, given its positive predictive value of 88% (95% CI, 76–95%) for (GAA)_≥250_-*FGF14* status and 89% (95% CI, 77–95%) for combined (GAA)_≥250_-*FGF14* and (GAA)_200-249_-*FGF14* statuses in our study.

#### Analysis of double-blind placebo-controlled clinical trial data

We next re-analysed placebo-controlled video-oculography data available for four patients with DBN—now known to be (GAA)_≥250_-*FGF14*-positive—from a previous randomised double-blind clinical trial.^17^ In addition to being double-blind, all trial assessments were blinded to the underlying GAA-*FGF14* genotype, which was not discovered until after the trial. The absolute value of the SPV decreased under treatment with 4-AP (mixed-effect analysis of repeated measures, *F*(2,6) = 6.31, *p* = 0.033), but not under placebo (mixed-effect analysis of repeated measures, *F*(2,5) = 0.45, *p* = 0.66) (Fig. 6c). The decrease in SPV was significant 60 min after administration of a single dose of 5 mg of 4-AP compared to intra-individual baseline measurements (least squares mean difference: 2.63°/s; 95% CI, 0.75–4.51; Fisher's least significant difference test, *p* = 0.014), and persisted under a stable dose of 40 mg of 4-AP daily (least squares mean difference: 1.97°/s; 95% CI: 0.08–3.84; Fisher's least significant difference test, *p* = 0.043). This effect corresponds to relative reductions (improvements) of SPV by 38–109% after a single dose, and 32–99% under stable treatment with 4-AP (Fig. 6d).

## Discussion

This study tested the hypotheses, and showed that *FGF14* (GAA)_≥250_ repeat expansions are a frequent monogenic cause of idiopathic DBN syndromes, a large proportion of patients with GAA-*FGF14* disease are responsive to 4-AP, and *FGF14* (GAA)_200-249_ alleles are potentially pathogenic. Our findings show that *FGF14* (GAA)_≥250_ repeat expansions account for almost 50% of cases of previously idiopathic DBN cases in a large cohort of European patients, thus corroborating the association of DBN with GAA-*FGF14* disease. While *FGF14* GAA repeat expansions have recently been shown to be a common cause of spinocerebellar ataxia (SCA27B)—which is associated with DBN in up to 40–60% of patients^1^^,^^3^^,^^4^^,^^24^—, the high frequency of *FGF14* (GAA)_≥250_ repeat expansions in patients with a non-ataxic DBN presentation was unexpected. One explanation may be that DBN is a milder endophenotype of GAA-*FGF14* disease, in which overt cerebellar ataxia and other multisystemic involvement can be absent (or, in some cases, develop later in the disease course). Our results further suggest that genetic testing for *FGF14* GAA repeat expansion should now become part of the diagnostic work-up of patients with idiopathic DBN, especially in the presence of additional cerebellar signs.

By screening a cohort of patients with idiopathic DBN, our study closes a gap in the delineation of the phenotypic spectrum and evolution of GAA-*FGF14* disease. The identification of additional floccular/parafloccular cerebellar ocular motor signs in all patients with DBN carrying an *FGF14* (GAA)_≥250_ expansion indicates that the basic dysfunction likely arises from this cerebellar region and that pure DBN is an uncommon manifestation in GAA-*FGF14* disease. Furthermore, the observation of DBN and other cerebellar ocular motor signs manifesting up to 8 years prior to the development of gait impairment suggests that such signs may present in isolation early in the disease course. However, the recurrent identification of *FGF14* expansions in patients with DBN plus additional isolated cerebellar ocular motor signs but without overt ataxia despite disease duration of up to 16 years raises the possibility that GAA-*FGF14* disease may remain limited to the cerebellar ocular motor system without broader cerebellar involvement in a subset of patients. This indicates that cerebellar ataxia may not be a universal feature of GAA-*FGF14* disease and that ocular motor signs and ataxia may each represent different features along a continuum of variable cerebellar involvement. Future natural history studies will be needed to determine whether cerebellar ataxia eventually develops in all patients. Nevertheless, the identification of a more limited phenotype in a sizeable number of patients suggests that the overall frequency of GAA-*FGF14* disease may even be higher than previously estimated.

While BVP has been suggested to be a recurrent feature of GAA-*FGF14* disease,^8^ our study provides an estimate of the frequency and temporal evolution of BVP in a large cohort of patients with GAA-*FGF14* disease. Ancillary vestibular assessment performed in more than 95% of patients carrying a (GAA)_≥250_ expansion documented BVP in 10% of them. BVP appears to remain relatively mild and be a late feature in GAA-*FGF14* disease compared to cerebellar ataxia, as it developed on average more than 10 years after disease onset.

Our study identified clinical features discriminating (GAA)_≥250_-*FGF14* from (GAA)_<200_-*FGF14* DBN syndromes. Of all clinical signs, impairment of the visual fixation suppression of the VOR was most strongly associated with GAA-*FGF14* disease, also indicating a dysfunction of the cerebellar flocculus/paraflocculus.^25^ While multiple areas of the brain are involved in gaze stabilization during head movement, the cerebellar flocculus/paraflocculus is critical in modulating the VOR.^25^ Early dysfunction of these two structures of the vestibulocerebellum, which are also implicated in the pathophysiology of DBN,^26^, ^27^, ^28^, ^29^ provides a unifying theory for the frequent and early co-occurrence of DBN, impaired VOR cancellation and other eye movement abnormalities, and vertiginous symptoms in GAA-*FGF14* disease.^30^

Functional impairment, as captured by the FARS functional disability stage and the need for walking aid, increased relatively slowly with disease duration in patients carrying a (GAA)_≥250_ expansion. This finding confirms previous reports^1^^,^^3^ and extends the evidence to a larger, independent group of patients with GAA-*FGF14* disease exhibiting a DBN phenotype. The slow accrual of disability was evidenced by an average increase of 0.10 FARS stage per year of disease and the need for mobility aids in 20% of patients.

The pathogenic potential of (GAA)_200-249_ alleles has recently been suggested based on the observation of segregation of (GAA)_200-249_ alleles and (GAA)_≥250_ expanded alleles with the disease in an affected family with autosomal dominant late-onset slowly progressive cerebellar ataxia.^19^ Extending this preliminary evidence, we now provide quantitative gene burden evidence for this notion by showing a significant enrichment of (GAA)_200-249_ alleles in patients with DBN compared to controls. In addition, we observed that impairment of the visual fixation suppression of the VOR and treatment response to 4-AP, both strongly associated with (GAA)_≥250_-*FGF14* status, were significantly more common in patients carrying a (GAA)_200-249_ allele than in patients carrying a (GAA)_<200_ allele (but not different from patients carrying a [GAA]_≥250_ expansion). It might thus be conceivable that *FGF14* (GAA)_200-249_ alleles may cause a milder cerebellar phenotype more commonly manifesting with isolated DBN and cerebellar ocular motor signs. Our findings align with those of a recent study, which found significant enrichment of (GAA)_180-249_ alleles in patients with ataxia compared to controls,^31^ yet diverge from another study reporting no enrichment of alleles with fewer than 300 GAA triplets in patients with ataxia compared to controls.^32^ This discrepancy may be partly explained by the inclusion of patients with hereditary ataxia of known cause in the latter study,^32^ potentially skewing the allele distribution toward smaller sizes. In comparison, our study analysed allele frequencies in a genetically unsolved cohort of patients with DBN and a substantially larger control population. Furthermore, our inclusion criterion likely resulted in an enrichment for larger *FGF14* alleles since DBN is commonly associated with GAA-*FGF14* disease.^24^ These observations underscore the need to conduct large-scale studies to re-evaluate the pathogenic threshold of GAA-*FGF14* disease.

Our study provides additional evidence for the symptomatic benefit of 4-AP in more than 50 patients with GAA-*FGF14* disease. Our findings showed that 4-AP treatment was associated with a clinician-reported treatment response in 80% and a patient-reported meaningful benefit in 59% of patients carrying a (GAA)_≥250_ or a (GAA)_200-249_ allele. In comparison, such treatment responses were significantly lower in patients carrying a (GAA)_<200_ allele (31% and 11%, respectively). Response to 4-AP in fact represented a strong predictor of (GAA)_≥250_-*FGF14* and (GAA)_200-249_-*FGF14* status in our study (positive predictive value, 89%).

Albeit still preliminary due to the small sample set, we present piloting double-blind, placebo-controlled randomised treatment data of 4-AP in four patients with GAA-*FGF14* disease.^17^ All four patients showed an improvement of SPV of the DBN on 4-AP, but not placebo. This observation bolsters the existing open-label real-world evidence for the treatment efficacy of 4-AP in GAA-*FGF14* disease observed in our current cohort and in previous cohorts.^3^^,^^9^ A larger randomised controlled trial is, nonetheless, warranted to confirm these findings. Our study highlights the potential of video-oculographic assessment of DBN as a responsive outcome measure for treatment. This approach could complement a promising battery of quantitative digital-motor outcomes,^9^ including sensor measures of gait and balance, in future trials of 4-AP for GAA-*FGF14* disease.

*FGF14* encodes the intracellular fibroblast growth factor 14 protein that is widely expressed throughout the central nervous system, most abundantly in the cerebellum.^33^ FGF14 regulates spontaneous and evoked firing of Purkinje cells by interacting with and modulating the function of voltage-gated sodium channels at the axon initial segment.^34^, ^35^, ^36^ Loss of FGF14 function in mice has been shown to attenuate repetitive firing of Purkinje cells as a result of impairment of sodium channel kinetics, ultimately leading to motor incoordination and imbalance.^37^, ^38^, ^39^ As the intronic *FGF14* GAA repeat expansion appears to cause loss of function of the gene,^1^ it may result in altered Purkinje cell excitability, potentially causing DBN, which is thought to arise from Purkinje cell hypofunction in the cerebellar flocculus.^27^, ^28^, ^29^^,^^40^ Furthermore, the mechanisms by which 4-AP, a voltage-gated potassium channel (K_v_) blocker,^41^ ameliorates symptoms in GAA-*FGF14* disease are yet to be established although it may involve restoration of cerebellar Purkinje cell rhythmic firing property, as shown in other forms of hereditary ataxia.^41^^,^^42^ 4-AP may thus compensate the reduced neuronal excitability and firing defects of Purkinje cells observed with loss of FGF14 function.^37^^,^^39^^,^^43^

The results of our study need to be interpreted in light of some limitations. First, multi-centre studies with cohorts from different ethnic backgrounds are needed to replicate our findings, which were drawn from a single-centre patient cohort of overwhelmingly European background. Second, the retrospective design of our study, which specifically focused on DBN (rather than ataxia) and functional staging of patients (using the FARS functional staging), limited our ability to use additional ataxia and non-ataxia severity scales, such as the Scale for Assessment and Rating of Ataxia (SARA)^44^ and the Inventory of Non-Ataxia Signs (INAS)^45^ since these were not systematically recorded during patient visits. Third, future prospective natural history studies will be required to accurately track the phenotypic evolution of GAA-*FGF14* disease from its earliest stage and to establish whether a subset of patients never develops overt cerebellar ataxia. Furthermore, since patients from our study did not undergo video-oculography, the frequency of some of the cerebellar ocular motor signs might have been underestimated due to the lower sensitivity of clinical examination compared to laboratory-based assessment. The incorporation of video-oculography in future studies may therefore allow for a better delineation of the ocular motor phenotype of GAA-*FGF14* disease. In addition, we were unable to ascertain whether the DBN was intensified in the head-down position as this maneuver was not routinely performed during patient's assessment. Fourth, our findings of potential pathogenicity of *FGF14* (GAA)_200-249_ alleles are largely preliminary and require validation through additional segregation studies, larger case–control series, and functional studies. Fifth, although we show a treatment effect of 4-AP in several outcomes, including clinician-reported and quantitative digital-motor outcomes, the benefits reported herein need validation in a larger randomised placebo-controlled trial. Sixth, the relatively small sample size of our study raises the possibility of spare-data bias, potentially affecting the precision and reliability of our effect size estimates. Seventh, our study design and the retrospective nature of data collection inherently limits our ability to identify and control for all potential confounding variables, possibly impacting the observed associations. Finally, while this study highlights the therapeutic potential of 4-AP in GAA-*FGF14* disease, the exact mechanisms by which 4-AP exerts its beneficial effects warrant further investigation.

In conclusion, we showed that GAA-*FGF14* disease is a highly frequent monogenic cause of DBN syndromes and that DBN presentations need to be recognised as a major endophenotypic cluster of this recently described neurodegenerative disease. Our study suggests that GAA-*FGF14* disease may present along a continuum of variable cerebellar involvement, with some patients exhibiting isolated cerebellar floccular/parafloccular ocular motor signs without overt ataxia. Moreover, our study also provides preliminary evidence that the molecular basis of GAA-*FGF14* disease may need to be extended, given the potential pathogenicity of *FGF14* (GAA)_200-249_ alleles. Finally, we presented open-label real-world as well as piloting placebo-controlled evidence for the treatment efficacy of 4-AP in GAA-*FGF14* disease, further paving the way toward clinical trials.

## Contributors

Design or conceptualization of the study: DP, CW, BB, MSt, MSy.

Acquisition of data: DP, FH, CW, MCD, AT, CA, MJD, AC, GDG, KMB, JC, DR, AMH, SZ, BB, MSt, MSy.

Analysis or interpretation of the data: DP, FH, CW, MCD, AT, CA, MJD, BB, MSt, MSy.

Drafting or revising the manuscript for intellectual content: DP, FH, CW, MCD, AT, CA, MJD, AC, GDG, KMB, JC, DR, AMH, SZ, BB, MSt, MSy.

DP, FH, CW, MCD, AT, BB, MSt, MSy directly accessed and verified the underlying data reported in the manuscript.

All authors read and approved the final version of the manuscript.

## Data sharing statement

Individual deidentified patient data may be shared at the request of any qualified investigator upon reasonable request. No consent for open sharing has been obtained.

## Declaration of interests

DP, FH, CW, MCD, AT, CA, MJD, AC, GDG, KMB, JC, AMH, and BB report no disclosures. DR has received grant/research support from Janssen and Lundbeck; he has served as a consultant or on advisory boards for AC Immune, Janssen, Roche and Rovi and he has served on speakers bureaus of Janssen and Pharmagenetix. He also received honoraria from Gerot Lannacher, Janssen and Pharmagenetix, and travel support from Angelini and Janssen, all unrelated to the present manuscript. SZ has received consultancy honoraria from Neurogene, Aeglea BioTherapeutics, Applied Therapeutics, and is an unpaid officer of the TGP foundation, all unrelated to the present manuscript. MSt is Joint Chief Editor of the Journal of Neurology, Editor in Chief of Frontiers of Neuro-otology and Section Editor of F1000. He has received speakers honoraria from Abbott, Auris Medical, Biogen, Eisai, Grünenthal, GSK, Henning Pharma, Interacoustics, J&J, MSD, NeuroUpdate, Otometrics, Pierre-Fabre, TEVA, UCB, and Viatris. He receives support for clinical studies from Decibel, U.S.A., Cure within Reach, U.S.A. and Heel, Germany. He distributes M-glasses and Positional vertigo App. He acts as a consultant for Abbott, AurisMedical, Bulbitec, Heel, IntraBio, Sensorion and Vertify. He is an investor and share-holder of IntraBio. All are unrelated to the present manuscript. MSy has received consultancy honoraria from Janssen, Ionis, Orphazyme, Servier, Reata, Biohaven, Zevra, Lilly, GenOrph, and AviadoBio, all unrelated to the present manuscript. MSy is planning a treatment trial of 4-AP in GAA-*FGF14* disease together with Solaxa Inc. as a sponsor, but has not received any type of honoraria or funding from Solaxa.
